# Elevated 18:0 lysophosphatidylcholine contributes to the development of pain in tissue injury

**DOI:** 10.1097/j.pain.0000000000002709

**Published:** 2022-06-07

**Authors:** Dominic Anthony Friston, Joshua Cuddihy, Jessica Souza Luiz, An Hoai Truong, Laptin Ho, Meirvaan Basra, Peter Santha, Orsolya Oszlacs, Joao de Sousa Valente, Tim Marczylo, Sini Junttila, Helen Laycock, Declan Collins, Marcela Vizcaychipi, Attila Gyenesei, Zoltan Takats, Gabor Jancso, Elizabeth Want, Istvan Nagy

**Affiliations:** a Nociception Group, Division of Anaesthetics, Pain Medicine and Intensive Care, Department of Surgery and Cancer, Imperial College London, London, United Kingdom; b Department of Anaesthetics, Chelsea and Westminster NHS Trust, London, United Kingdom; c Department of Physiology, University of Szeged, Szeged, Hungary; d Section of Vascular Biology and Inflammation, School of Cardiovascular Medicine and Research, BHF Cardiovascular Centre of Research Excellence, King's College London, London, United Kingdom; e UK Health Security Agency, Radiation, Chemical and Environmental Hazards, Didcot, United Kingdom; f Turku Bioscience Centre, University of Turku, Turku, Finland; g Szentagothai Research Centre, University of Pecs, Pécs, Hungary; h Biomolecular Medicine, Department of Metabolism, Digestion and Reproduction, Imperial College London, London, United Kingdom

**Keywords:** Primary sensory neurons, TRPV1, TRPV2, Mechanical allodynia, Heat hyperalgesia, Burn injury

## Abstract

Tissue injuries, including burns, are major causes of death and morbidity worldwide. These injuries result in the release of intracellular molecules and subsequent inflammatory reactions, changing the tissues' chemical milieu and leading to the development of persistent pain through activating pain-sensing primary sensory neurons. However, the majority of pain-inducing agents in injured tissues are unknown. Here, we report that, amongst other important metabolite changes, lysophosphatidylcholines (LPCs) including 18:0 LPC exhibit significant and consistent local burn injury-induced changes in concentration. 18:0 LPC induces immediate pain and the development of hypersensitivities to mechanical and heat stimuli through molecules including the transient receptor potential ion channel, vanilloid subfamily, member 1, and member 2 at least partly via increasing lateral pressure in the membrane. As levels of LPCs including 18:0 LPC increase in other tissue injuries, our data reveal a novel role for these lipids in injury-associated pain. These findings have high potential to improve patient care.

## Abstract 2

Supplemental Digital Content is Available in the Text.

18:0 lysophosphatidylcholine exhibits up-regulation in burn tissues and, through modifying the lateral pressure in primary sensory neurons, contributes to pain in burn injury.

## 1. Introduction

Injuries such as burn injury induce inflammation to restore tissue integrity and function.^18,42^ This inflammatory reaction is associated with the development of pain, which is initiated and maintained by agents accumulating in the injured and inflamed tissue and acting on nociceptive primary sensory neurons.^18,19,21,42^ Sustained activation of primary sensory neurons, through complex nociceptive mechanisms, can lead to the development of chronic pain.^18,40,42,78^ The majority of studies have measured agents associated with tissue injuries in circulation. Consequently, the identity and importance of locally acting agents remain largely unknown.^24,25,42,69,79^ Hence, comprehensive analysis of local agents in injured/inflamed tissues will further our understanding of both inflammatory processes and peripheral mechanisms of nociceptive processing.

Recently, we explored metabolites in dermal microdialysates obtained from rat skin collected during the first 3 hours of a burn injury model.^25^ However, the supervised partial least squares discriminant analysis employed could not account for the data's temporal arrangement and may not have detected important metabolites altered, for example, more transiently post injury.^25^ To elucidate this potential multidimensionality of the burn effect, we employed canonical variates analysis (CVA) for discriminant analysis to the previously acquired ultraperformance liquid chromatography–mass spectrometry (UPLC-MS) data set.^60^

Here, we report that the analysis identified the presence of various species of lysophosphatidylcholines (LPC) including 18:0 LPC exhibiting a consistent increase in the microdiasylates following burn injury. Furthermore, we also report that findings from in vitro and in vivo studies indicate that 18:0 LPC significantly contributes to the development and persistence of pain, at least partly, through modifying the lateral pressure in the cytoplasmic membrane to activate, most likely among other channels, the transient receptor potential ion channels, vanilloid subfamily, member 1 (TRPV1), and member 2 (TRPV2) ion channels.

## 2. Methods

### 2.1. Ethics

This study was performed in accordance with the United Kingdom Animals (Scientific Procedures) Act 1986, the revised National Institutes of Health *Guide for the Care and Use of Laboratory Animals*, the Directive 2010/63/EU of the European Parliament and the Council on the Protection of Animals Used for Scientific Purposes and the guidelines of the Committee for Research and Ethical Issues of IASP published in PAIN, 16 (1983) 109 to 110. Good Laboratory Practice and ARRIVE guidelines were observed, and all animal procedures were approved by veterinary services (Central Biological Services) at Imperial College London, United Kingdom. Collection and use of human skin samples for this study were approved by the Health Research Authority London—Westminster Research Ethics Committee (IRAS reference: 189005, REC:16/LO/0203).

### 2.2. Human biopsy

Four patients with dermal scalding–type burn injuries requiring surgical wound debridement and split skin grafting were recruited and consented. Five to 9 days after injury, while anaesthetised with either local or general anaesthetics as part of their usual care, three 3-mm punch biopsy samples were taken during surgery: 2 samples from the burn wound and 1 sample from a nonburned area of skin used as the donor site for skin grafting. Tissue samples were stored at −80°C until cryosectioning.

### 2.3. Burn injury model

Sprague Dawley male rats (125-250 g) were housed in 20 to 22°C climate-controlled rooms on a 12-hour light–dark cycle with free access to food and water. Anaesthesia was induced with 1.5 g/kg urethane injection (intraperitoneal [i.p.]) when animals were used for microdialysis. For the chlorpromazine study, rats were anaesthetised with 2% to 3% isoflurane. Body temperature was controlled to 37°C with a heat blanket, rectal thermometer, and homeothermic control unit (50-7061-F; Harvard Apparatus, Cambridge, United Kingdom). Deep partial thickness scald injury was induced, as described previously.^24,25,79,89^ Briefly, the burn was administered to 1 hind paw by a 2-minute submersion to the ankle in 60°C saline. Simultaneously, the contralateral paw was submerged in room temperature saline as a control for fluid immersion.

In the chlorpromazine study, chlorpromazine (50 mg/kg i.p.) was administered 10 minutes after the onset of injury, and the contralateral paw was not submerged. Animals were terminally anaesthetised at 60 minutes using pentobarbital (0.3 mg/kg i.p). Animals were perfused with saline followed by 4% paraformaldehyde. The spinal cord (L4-L5) segments were dissected and cryoprotected in 30% sucrose.

### 2.4. Microdialysis

Microdialysis was conducted as described previously.^25^ Briefly, sterile linear microdialysis probes (400 μm 3 MDa cutoff microdialysis catheters; Dermal Dialysis, Erlangen, Germany) were placed in the dorsal aspect subcutaneous tissue of both hind limbs at the dermal border via a 25-G needle with an active uptake distance of 10 mm 50 minutes prior to the induction of the burn injury. Probes were perfused with Ringer's solution (Baxter, Newbury, United Kingdom) at 2 μL/minute, and microdialysate was collected in 30 minute fraction 30 minutes before, and for 3 hours following burn injury. Following terminal anaesthesia by i.p. sodium pentobarbital (0.3 mg/kg), the dialysed tissues were excised for histological verification of probe depth^24^ or RNA extraction.

### 2.5. Metabolomics

Microdialysate fractions were diluted 1:3 in −20°C methanol (MeOH), vortexed for 20 seconds, stored at −20°C for 20 minutes, and centrifuged (12,000 × *g*, 10 minutes) to precipitate protein. Resulting supernatants were dried via vacuum evaporation (Eppendorf Concentrator Plus ‘Speed Vac’; V-AQ mode, 45°C, 2 hours) before resuspension in water (LC-MS grade; Sigma-Aldrich, Gillingham, United Kingdom). Quality control (QC) preparations consisted of pooling 5 μL taken from every microdialysate fraction.

Metabolomics was conducted with an Acquity UPLC System, a HSS T3 column (1.8 μm, 100 × 2.1 mm; Waters, Manchester, United Kingdom) and a Synapt G2-S Q-ToF (Waters) in ESI^+^ and ESI^−^ modes.^25^ A 12-minute chromatographic gradient was employed with a flow rate of 0.4 mL/minute (Supplemental Table 1 for parameters, available at http://links.lww.com/PAIN/B664). LockSpray was acquired using leucine enkephalin (0.2 ng/μL in 50:50 acetonitrile:water). Mass spectrometry acquisition was in sensitivity mode. Centroid mode data were acquired between *m*/*z* 100 to 1200 with a scan time and interscan delay of 100 and 10 milliseconds, respectively. The system was calibrated with sodium formate and conditioned with 10 injections of the QC prior to sample analysis, in which run order was randomised. Quality control samples were injected every 5 samples to assess instrument stability and run reproducibility. Following data analysis (Data analysis section in Methods), tandem mass spectrometry (UPLC-MS/MS) was used for compound identification. Aside from a collision energy ramp of 10 to 40 V, the same system and parameters were used as for initial exploratory mass spectrometry. Structures were confirmed by the identification of shared parent and fragment *m*/*z* ratios and retention time between the endogenous metabolite feature and a standard solution. Standard solutions were prepared from >99% purity powdered 14:0 LPC, 16:0 LPC, and 18:0 LPC (Avanti Polar Lipids, Birmingham, AL) for injection at 10 μg/mL in 50:50 water/MeOH.

### 2.6. Lipidomics

Human tissue samples were thawed on ice and weighed, then transferred into bead-beater tubes containing 1-mm zirconium beads and 0.75 mL 1:1 methanol:water. Samples were homogenised in a Precellys bead beater for 6 cycles of 40 seconds at 6500 Hz, cooling on ice in between cycles. Subsequently, bead-beater tubes were centrifuged for 10 minutes at 10,000*g* and 4°C. Supernatant was stored for polar metabolite analysis, and the pellet further extracted by adding 0.75 mL 3:1 dichloromethane:methanol solution. The mixture underwent 6 further cycles in the Precellys bead beater (40 seconds at 6500 Hz), followed by centrifugation (10 minutes at 10,000*g* and 4°C). Lipid-rich supernatant was transferred to MS vials, which dried overnight in a fume cupboard. After 24 h, the dry sample was resuspended in 80 μL 1:1 methanol:water, vortexed (30 seconds), sonicated (30 minutes), and injected onto the UPLC-MS system. Quality control samples, prepared by pooling 10 μL of each human sample (burn and control), were used to condition the column and analysed every 5 samples to assess instrument stability and reproducibility.

An UPLC Acquity system (Waters, Manchester, United Kingdom) equipped with CSH column (2.1 × 100 mm, 1.7 μm—Waters) was used for reversed-phase chromatography as follows: flow rate 400 μL/minute, column temperature 55°C, gradient time 20 minutes.^33^ Mobile phase composition comprised the following: (A) acetonitrile/water (60:40) + 10 mM ammonium formate + 0.1% formic acid, (B) isopropanol/acetonitrile (90:10) + 10 mM ammonium formate + 0.1% formic acid. Ultra performance liquid chromatography was coupled with a Synapt G2-S QToF MS (Waters) operating in centroid mode for untargeted analysis. In both ionisation modes, we used a cone voltage of 30 kV, capillary voltage of 2 kV, and scan range 50 to 2000 Da. Settings for ESI^+^ were as follows: source temperature of 120°C, desolvation temperature of 600°C, desolvation gas flow of 1000 L/hour, and cone gas flow of 150 L/hour. For ESI^−^, these selected parameters were 100°C, 450°C, 600 L/hour, and of 50 L/hour, respectively.

### 2.7. Desorption electrospray ionisation mass spectrometry imaging

Human skin specimens were embedded in a (hydroxypropyl)-methylcellulose/polyvinylpyrrolidone hydrogel, sectioned at 10-µm thickness and mounted on glass slides. Desorption electrospray ionisation mass spectrometry (DESI-MS) imaging was performed on sections of burned and nonburned skin from patients in positive and negative ion mode using a Waters Xevo X2 QTOF instrument (Waters). Analysis was performed between 50 and 1500 *m*/*z* using a line-by-line sampling method using pre-set *x* and *y* coordinates. A spatial resolution of 50 µm by 50 µm was used for all experiments. All tissue sections were subsequently subjected to haematoxylin and eosin staining. Annotated haematoxylin and eosin images were coregistered with the mass spectrometry imaging data to match metabolite distributions with the underlying tissue morphology. To correct for mass shifts, mass spectrometry imaging data were lockmass corrected to raffinose, which was doped into the sprayer solvent in a concentration of 10 ppm. Corrected files were uploaded into an in-house–developed Matlab (Mathworks, Cambridge, United Kingdom) toolbox for processing.

### 2.8. RNA extraction and next-generation sequencing

Following excision, dialysed tissue was stored in RNAlater. Tissues were homogenised with a rotor-stator mixer and total RNA extracted with RNeasy Fibrous Tissue Mini and Midi kits (Qiagen, Manchester, United Kingdom) according to the manufacturer's protocols. Next-Generation Sequencing libraries were prepared via poly-A enrichment (New England Biolabs, Ipswich, MA) according to the manufacturer's protocol. Samples were sequenced via Illumina HiSeq2500 by 50 base pair single-end reads on 1 lane.

### 2.9. Culturing primary sensory neurons

Dorsal root ganglia (DRG) from male Sprague Dawley rats (125-200 g) and wild-type (WT) and TRPV1^−/−^ C57/BL6j mice were excised and transferred into DMEM/F12 (Sigma). Tissue was digested at 37°C in 0.125% collagenase (Lorne Diagnostics, Reading, United Kingdom) and 0.05% trypsin-EDTA (Thermo Fisher Scientific, Dartford, United Kingdom) for 3 hours and 5 minutes, respectively, before trituration and centrifugation through a 1-mL bovine serum albumin cushion at 200 × g for debris removal. Cells were resuspended and plated on poly-dl-ornithine (Sigma)–coated glass coverslips in DMEM (Sigma-Aldrich, Gillingham, United Kingdom) with 50 IU/mL penicillin (Thermo Fisher Scientific), 50 ug/mL streptomycin, 2% Ultroser G (Sartorius, Goettingen, Germany), 2 mM l-glutamine (Thermo Fisher Scientific), and 50 ng/mL mouse 2.5 S nerve growth factor (Promega, Southampton, United Kingdom). Cells were incubated at 37°C and 5% CO_2_ for 1 day prior to calcium imaging.

### 2.10. Culturing and transfecting HEK cells

HEK293T cells were incubated at 37°C and 5% CO2 in DMEM (Sigma) with 2% foetal bovine serum (Sigma), 50 IU/mL penicillin (Thermo Fisher Scientific), 50 μg/mL streptomycin, and 2 mM l-glutamine (Thermo Fisher Scientific) and passaged to poly-dl-ornithine (Sigma)–coated glass coverslips for calcium imaging. Transfection began at least 8 hours after plating with PLUS Reagent and lipofectamine 2000 (Thermo Fisher Scientific) according to the manufacturer's protocol. In brief, a transfection medium was prepared by 15-minute room temperature incubation of 25 μL DMEM with 0.2 μg of TRPV1 and eGFP cDNA and 1 μL PLUS Reagent (Thermo Fisher Scientific) before addition of 25 μL DMEM with 1 μL lipofectamine 2000 (Thermo Fisher Scientific) for a further 15 minutes per well. Then, 50 μL of the preparation was added to each well, containing HEK293T cells in 200 μL DMEM. Cells were used 24 hours later.

### 2.11. Ratiometric Ca2+ imaging

HEK293T and DRG cells were loaded with 100 nM Fura-2 AM (Sigma) for 40 minutes before washing with extracellular fluid (in mM: NaCl 150, KCl 5, MgCl_2_ 2, CaCl_2_ 2, HEPES 10, glucose 10; pH 7.4). Coverslips were mounted on a Nikon Eclipse TE300 microscope with an optiMOS Scientific CMOS Camera (Teledyne QImaging, Birmingham, United Kingdom), and images acquired with Winfluor (Version 3.7.3; University of Strathclyde, Glasgow, United Kingdom) every 2 seconds with excitation at 355 and 380 nm and emission recorded at 510 nm. Control buffer and drugs were perfused via a 6-way gravity flow system (∼600 μL/minute). The outflow was placed ∼100 to 200 μm from the group of cells from which the recordings were made. Recordings were performed at 37°C, except where the effect of heat stimulation was assessed when the temperature of the superfusate was increased from 32 to 55°C linearly. The temperature of the superfusate was continuously measured at the opening of the superfusing tube and recorded alongside measuring fluorescent intensities.

### 2.12. Whole-cell voltage clamp recordings

Whole-cell voltage clamp recordings were collected from cultured rat primary sensory neurons using 2 to 4 MΩ patch pipettes pulled from borosilicate glass capillaries. Recordings were obtained with an Axopatch 200B amplifier, Digidata 1200 digitizer (Molecular Devices, San Jose, CA), and Clampex (pClamp 8.2 software package, Molecular Devices, San Jose, CA). Whole-cell currents were recorded at 10 kHz with 2 kHz filtering with >60% compensation of the capacitance and series resistance. Cells were held at −60 mV, and 10-second 30 nM capsaicin pulses were applied.

### 2.13. In vivo experiments

Nociceptive withdrawal response to radiant heat stimulation was studied using the Hargreaves method. Male Sprague Dawley rats (250-300 g) were placed on a glass surface in a transparent plastic cage and allowed to adapt to their environment for 15 minutes before testing. The heat stimulus was directed onto the plantar surface of the hind paw using a Hargreaves apparatus (Ugo Basile, Gemonio, Italy) calibrated to give a withdrawal latency of ∼10 seconds in control animals. To prevent tissue damage, a cutoff time of 20 seconds was chosen. Withdrawal latencies were measured 3 times on both hind paws of each rat. The average of these values was defined as the basal withdrawal latency. After intracutaneous administration of 0.4% 18:0 LPC (100 µL) into the plantar skin of the right paw, withdrawal latencies were determined at 30, 60, 90, and 120 minutes after injection.

Changes in mechanical nociceptive thresholds were measured on both hind paws using a Dynamic Plantar Aesthesiometer (Ugo Basile). Rats were placed in closed perspex cages on a metal mesh surface. After a short adaptation period, the stimulator unit was placed below the plantar surface of the hind paw, and the metal test filament positioned toward the mid portion of the sole, and a force of 0 to 50 g for 10 seconds was applied. The procedure was repeated 3 times, and withdrawal responses were averaged. Mechanical threshold was determined both before (basal mechanical threshold) and 30, 60, 90, and 120 minutes after the injection of 0.4% 18:0 LPC (100 µL) into the plantar skin of the right hind paw.

In animals pretreated with capsazepine or tranilast (Tocris, Abingdon, United Kingdom), respectively, 100 µL of 10 mM capsazepine or 600 µM tranilast were injected into the plantar skin of the right hind paw 15 minutes before the injection of 0.4% 18:0 LPC.

### 2.14. Immunostaining

Cryoprotected tissues were cut into 15-μm-thick sections, which were placed on glass slides. Sections were incubated in phosphate-buffered saline containing 0.3% Triton-X-100 then in 10% normal donkey serum (Jackson ImmunoResearch Labs, Ely, United Kingdom). Sections were then incubated in anti-p-S10H3 antibody (PA5-17869, Thermo Fisher Scientific) overnight in a humidification chamber prior to incubation with secondary antibodies (AlexaFluor TM568 Donkey Anti-Rabbit IgG H + L, A21206, Thermo Fisher Scientific, Dartford, United Kingdom). Sections were mounted with diamidino-2-phenylindole–containing mounting medium (ProLong Gold antifade, Thermo Fisher) and studied using an epifluorescent microscope (Lieca, Glen Urquhart, United Kingdom) equipped with a digital camera connected to a PC. The number of fluorescent cells was counted.

### 2.15. Data analysis and statistics

#### 2.15.1. Data analysis was performed blinded

##### 2.15.1.1. Metabolomics

Canonical variates analysis (as implemented in Statistical Parametric Mapping academic freeware for Matlab) was used to test for effects of the burn within the highly multivariate data generated via UPLC-MS by identifying (orthogonal) mixtures of dependent variables that correlate with (orthogonal) mixtures of independent variables. Detailed description of CVA is provided in figure legend for Supplemental Figure 1 (available at http://links.lww.com/PAIN/B664). The top 5% of the features were considered for structural elucidation by MS/MS; targets were selected on the basis of canonical weight, signal strength, and potential structures sourced from the Human Metabolome Databse (HMDB)^90,91^ as determined by *m*/*z* ratio and any fragmentation visible in the MS analysis.

##### 2.15.1.2. Lipidomics

Raw data files were assessed in MassLynx v4.1 software (Waters), converted to mzXML format using ProteoWizard,^13^ and sequentially uploaded to XCMS Online^73^ for data preprocessing. A table was generated containing the *m*/*z* ratio, retention time, and peak area intensity values for each detected feature for all human samples evaluated. Prior to statistical analysis, feature intensities were normalised by sample weight. For QC samples, the arithmetic mean weight of all the mixed samples was used. A threshold of 30% coefficient of variation was set for all detected metabolite features in the QC samples—any feature above this threshold was discarded before statistical analysis because it was not considered reliable. Data underwent further normalisation by median (row wise) transformation by generalised logarithm and auto scaling (column wise). Additionally, variances within groups were mathematically tested and inequality was confirmed. Unsupervised principal components analysis was performed. For univariate analysis, unpaired Welch *t* tests were calculated for all features. The Holm–Bonferroni method was used to correct for false discovery. Statistical significance was considered for features with q < 0.05.

Significant features were progressed for putative identification using HMDB,^92^ Lipid Maps,^22^ and METLIN.^27^ For negative ionisation mode, only M-H adducts were searched, with a tolerance of ±0.05 Da. For positively ionised molecules, both M + H and M + Na^+^ adducts were searched with the same mass variation tolerance. Lipids with delta <35 ppm between observed and expected *m*/*z* values were considered promising for annotation.

##### 2.15.1.3. DESI-MS

A data set of 72 individual samples (36 paired burn and control) was preprocessed to control for mass error and drift/shift between experiments and allow comparisons between all annotated samples. Whole-tissue annotation was performed to include all tissue pixels from burn and control skin, respectively. A mean average of spectra across all pixels within a sample was generated and compared against corresponding samples in the data set. To allow for paired analysis, each sample was linked to the patient it came from. Univariate analysis using Kruskal–Wallis one-way analysis of variance (ANOVA) was performed on all variables within the data set extracted with mean intensity, fold change, *P* value (*t* test), and q values (Benjamini–Hochbach–Yeuketeli method for false detection rate correction), and fold-change value generated. Data were analysed using purpose built in-house software in Matlab (Mathworks).

##### 2.15.1.4. RNA sequencing

Data were analysed by sequencing reads mapped to the *Rattus norvegicus* Rnor5.0 reference genome release using TopHat (version 2.0.9).^37^ Gene-wise read counts were measured using HTSeq (version 0.5.4p3)^4^ and normalised via TMM normalization as implemented in the edgeR package in R (R version 3.0.1, Bioconductor version 2.12).^50,63^ For statistical analysis, data were voom transformed, and differential expression between burn and control samples was detected using the Limma package in R.^62^ The threshold for differential gene expression was set at |FC| > 2 and FDR < 0.05. Enrichment analyses were conducted using topGO^3^ and gage packages in R. The present RNA-seq data were submitted to GEO with accession GSE102811.^7,20^

##### 2.15.1.5. Ca^2+^ imaging

Data were analysed offline with WinFlour and Clampfit (Molecular Devices) software packages. Briefly, after establishing regions of interest in the images, the time course of changes in fluorescent ratios were examined and exported to Clampfit, where SD of the baseline activity and maximum amplitudes of responses were measured. A change in the fluorescent ratio was accepted as a response if the generation of the response corresponded to the drug application and the amplitude of the change was >3 times the SD of the baseline activity. The number of cells in various groups and amplitude of responses of cells from at least 3 independent cultures were pooled and averaged. Cells not responding to the final depolarising stimulus were excluded from analysis.

The heat threshold of heat-responding cells was determined using temperature—ΔF_355_/F_380_ plots. Data of the plots exhibiting “accelerating” increase in [Ca^2+^]_i_ were used to generate Arrhenius plots.^86^ The cross section of extrapolated data of [Ca^2+^]_i_ below and above the threshold was determined in each cell and taken as heat threshold.

Normal distribution of data was assessed using the Shapiro–Wilk test. Significant change in the number of responding cells was assessed using Fischer exact test. Amplitudes and area under the curves (AUCs) of calcium transients were compared using the Student *t* test, ANOVA or 2-way repeated-measures ANOVA followed by Bonferroni or Tukey post hoc tests as appropriate.

##### 2.15.1.6. Behavioural

Repeated measurement two-way mixed design ANOVA was used for statistical analyses. In all groups, normality was checked by the Shapiro–Wilk test and homogeneity of variances was confirmed by Levene test in advance of performing the two-way mixed design ANOVA. Post hoc comparisons were performed using Tukey test.

##### 2.15.1.7. Immunostaining

Images were analysed by counting the number of fluorescent cells on images. A Shapiro–Wilk test was used to guide statistical analysis (alpha level 0.05). All results were parametric; hence, a Student *t* test of the mean between chlorpromazine treated and untreated groups was performed.

Differences in all experiments were regarded significant when *P* < 0.05 or q < 0.05 after false discovery rate correction. All data are expressed as mean ± SEM.

## 3. Results

### 3.1. Burn increases the lysophosphatidylcholine content in rat skin

Canonical variates analysis identified significant post injury burn effects in both positive electrospray ionization (ESI^+^) and ESI^−^ UPLC-MS data. It revealed increasing then maintained differences between levels of metabolites collected from burn tissues and contralateral naive tissues over time (Supplemental Figs. 1 and 2, available at http://links.lww.com/PAIN/B664), as represented by their respective canonical contrasts. After ranking 42,364 ESI^+^ and 13,829 ESI^−^ metabolite features by canonical weight, a measure of each individual metabolite feature's contribution to a canonical contrast, we explored the top 5% differentiating metabolite features in each ionization mode. Of these, 84 features demonstrating a burn-specific change in concentration were selected for structural identification.

**Figure 1.**
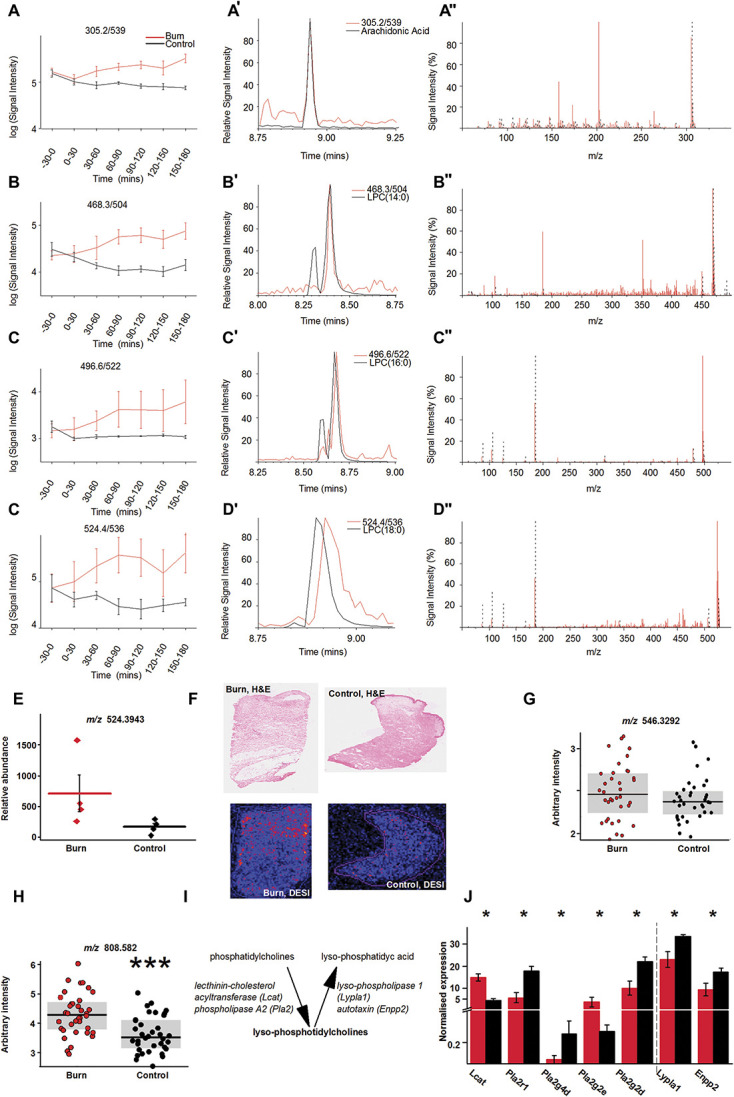
18:0 LPC exhibits increase in the burn skin. (A–D) Time-dependent change of the 4 most significantly and consistently increased metabolic features following burn injury in rats. (A′–D′) and (A″–D″) Identification of the metabolites, whose time-dependent changes are shown in (A–D); (A′–D′) show retention time, whereas (A″–D″) fragmentation profile, comparison of respective metabolites with standards. (E) Relative abundance of the analyte with *m*/*z* 524.3943, as a potential match for 18:0 LPC+H^+^, in biopsies from burn and nonburn skin sites (Student *t* test, *P* = 0.062). (F) Paired haematoxylin and eosin–stained (H&E) skin sections and DESI mass spectrometry image (DESI) for burn and control 10-μm sections from same patient. Desorption electrospray ionisation mass spectrometry imaging showing intensity and distribution of *m*/*z* 546.329, a potential match for 18:0 LPC+Na^+^. (G) Arbitrary intensity of *m*/*z* 546.329 in 36 patients in control and burn skin samples; grey box indicates interquartile range. (H) Arbitrary intensity of 808.582, a potential match for 18:0/18:2 phosphatidylcholine, in 36 patients in control and burn skin samples; grey box indicates interquartile range. (I, J) RNA-seq analysis of genes, whose products are involved in LPCs' metabolism. *P < 0.05; ***P < 0.0005. LPC, lysophosphatidylcholine; RNA-seq, RNA sequencing.

As previously identified in these data with partial least squares discriminant analysis, CVA detected ESI^+^ *m*/*z*/*RT* 123.1/83 (identified as niacinamide) and ESI^−^ *m*/*z*/*RT* 167/85 (identified as uric acid) as highly differentiating features with canonical weights of 0.011 and 0.0193, respectively.^25^ In addition, selected metabolite features included ESI^+^ *m*/*z*/*RT*, 137.0/198, 305.2/539, 468.3/504, 496.6/522, and 524.4/536 with canonical weights of 0.0172, 0.0109, 0.0148, 0.012, and 0.017, respectively (Figs. 1A–D; Supplemental Fig. 3, available at http://links.lww.com/PAIN/B664). Fragmentation profiles of *m*/*z*/*RT* 468.3/504, 496.6/522, and 524.4/536 were identical to those of 14:0 LPC (Figs. 1B–B″), 16:0 LPC (Figs. 1C–C″), and 18:0 LPC (Figs. 1D–D″), respectively, as confirmed with concomitant UPLC-MS/MS analyses of authentic standard preparations and fragmentation spectra in the HMDB^90,91^ and Metlin^67^ databases. The identity of other selected features (*m*/*z*/*RT* 137/198 and *m*/*z*/*RT* 305.2/539) could not be confirmed (Figs. 1A–A″ and Supplemental Fig. 3, available at http://links.lww.com/PAIN/B664).

### 3.2. Burn increases 18:0 LPC in human skin

In addition to acting as potent proinflammatory molecules, the 3 identified LPCs may further be particularly relevant in the context of pain because they can be hydrolysed to the algogenic lysophosphatidic acids (LPA) and thereby induce neuropathic pain through demyelination and activate various pain-related molecules expressed on primary sensory neurons.^5,15,32,41,44–46,53,54,61,64,74,81,87^ To further corroborate LPC's possible role in burn injury and associated pain, we investigated the presence of 14:0 LPC, 16:0 LPC, and 18:0 LPC in data generated through untargeted UPLC-MS lipidomics on human skin biopsies collected from burn and nonburn areas of 4 consented patients 5 to 9 days after scalding-type burn injury during debridement. We found a significant alteration of the lipid content of burn skin in general (Supplemental Fig. 4, available at http://links.lww.com/PAIN/B664) as well as evidence for the presence of *m*/*z* 468.3291, *m*/*z* 490.3041, *m*/*z* 496.3449, *m*/*z* 518.3257, and *m*/*z* 524.3943, respective likely matches for 14:0 LPC+H^+^, 14:0 LPC+Na^+^, 16:0 LPC+H^+^, 16:0 LPC+Na^+^, and 18:0 LPC+H^+^. Abundance analysis showed that among those, only *m*/*z* 524.3943 (18:0 LPC+H^+^) exhibited an increase in burn samples (Fig. 1E). Although the abundance of *m*/*z* 524.3943 was > five-fold higher in burn than control samples because of the small sample size and large variance within the data, the difference did not reach the level of significance (Student *t* test, *P* = 0.062, Fig. 1E).

Next, we investigated the tissue distribution of 18:0 LPC prepared from human skin biopsy sections by DESI-MS imaging. We found evidence for the presence of *m*/*z* 546.329, a potential match for 18:0 LPC + Na^+^ (Fig. 1F). Desorption electrospray ionisation mass spectrometry images showed that the upper and middle thirds of the dermis exhibited increased 18:0 LPC + Na^+^ concentration (Fig. 1F). However, quantification of the abundances showed that the change in 18:0 LPC + Na^+^ was not significant when all samples were assessed (Fig. 1G; q = 0.213, n = 38).

To explore these findings further, we analysed changes in human skin levels of 18:0/18:2 phosphatidylcholine (PC; *m*/*z* 808.582 = 18:0/18:2 PC + Na^+^), an immediate precursor for 18:0 LPC. We found that *m*/*z* 808.582 was significantly increased in burn samples when compared with control samples (Fig. 1H and Supplemental Fig. 5, available at http://links.lww.com/PAIN/B664, Student *t* test, *P* < 0.0001).

### 3.3. Burn induces changes in transcripts for enzymes involved in LPC metabolism

To further support LPC enrichment of burn skin, we analysed RNA-seq data of naive and burned rat skin^24^ (GEO with accession GSE102811) and determined that several of LPC's metabolic enzymes are differentially expressed. mRNA levels of LPC-synthesising lecithin-cholesterol acyltransferase and phospholipase A2 (Pla2) group 2 E (Pla2g2e) were significantly increased in burn skin (Figs. 1I and J), while Pla2r1, Pla2g4d, and Pla2g2d expression exhibited postburn down-regulation (Fig. 1J). mRNA expression of the 2 main degrading enzymes, autotaxin, and lysophospholipase 1 were also down-regulated in burn tissue (Fig. 1J).

Although *m*/*z* values of possible matches for 14:0, 16:0, and 18:0 LPCs were found in human tissues, only the abundance of 18:0 LPC exhibited an increase. While that increase did not reach statistical significance, the context of 18:0 LPC's significant elevation in animal studies, the significant increase of precursor levels in humans, and the evident transcriptional changes occurring in LPC's metabolic enzymes post burn motivated us to further investigate the role of 18:0 LPC in pain.

### 3.4. Subcutaneous 18:0 LPC injection induces pain-related behaviour

**Figure 2.**
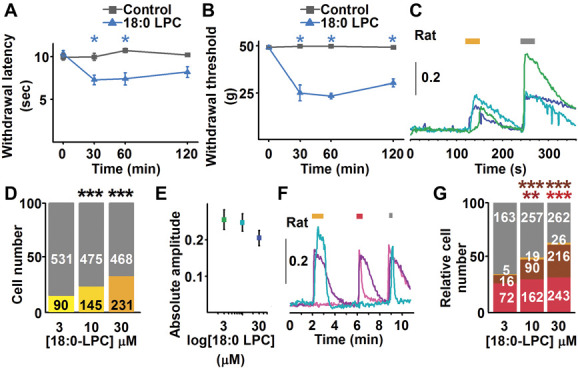
18:0 LPC induces pain-related behavior through activating a major group of nociceptive primary sensory neurons. (A and B) 18:0 LPC injected intraplantarly (0.4 mg/mL) induces the development of hypersensitivity to heat (A) and mechanical stimuli (B). (C) Superfusing 18:0 LPC (3, 10, 30 μM; yellow bar) induces an increase in intracellular calcium concentration in cultured rat primary sensory neurons. Grey bar indicates the application of 60 mM KCl. (D) The proportion of 18:0 LPC-responding cells exhibits a concentration-dependent increase. Asterisks indicate significant differences. (E) Absolute amplitude of the 18:0 LPC-evoked calcium transients does not exhibit concentration dependence. (F) The great majority of 18:0 LPC-responding rat cultured primary sensory neurons also respond to 500 nM capsaicin (purple trace). However, not all capsaicin-responding cells respond to 18:0 LPC (pink trace) and a small number of 18:0 LPC-responding cells do not respond to capsaicin (azure trace). Yellow, red, and grey bars above the recordings indicate 18:0 LPC (30 μM), capsaicin (500 nM), and KCl (60 mM) application. (G) Proportion of 18:0 LPC only (yellow), 18:0 LPC and capsaicin (brown), capsaicin only (red), and KCl only (grey) responding cells at 3 to 30 μM 18:0 LPC. Increasing the 18:0 LPC concentration alongside of increasing 18:0 LPC-responding neurons also increases the overall number of capsaicin-responding cells. *P < 0.05; **P < 0.001; ***P < 0.0001. LPC, lysophosphatidylcholine.

We injected 4 mg/mL (∼8 μM) 18:0 LPC subcutaneously into the dorsum of the rat paw, which induced an immediate and lasting nocifensive behaviour (licking, biting, shaking) and rapid development of oedema. While a ten-fold reduction of the 18:0 LPC dose (0.4 mg/mL; ∼800 nM) did not induce nocifensive behaviour or oedema, it led to the development of both mechanical allodynia and heat hyperalgesia, which persisted throughout the entire assessment period (repeated measurement two-way mixed design ANOVA, Tukey post hoc test; n = 6, *P* < 0.05 both for mechanical and thermal sensitivity; Figs. 2A and B). These findings suggest that at high doses 18:0 LPC immediately activates a group of nociceptive primary sensory neurons to induce nocifensive behaviour, whereas at lower doses, 18:0 LPC induces sensitisation resulting in the development of hypersensitivities to heat and mechanical stimuli.

### 3.5. 18:0 lysophosphatidylcholine activates nociceptive primary sensory neurons

To investigate the immediate activating effects, we assessed 18:0 LPC-evoked changes in intracellular calcium concentration ([Ca^2+^]_i_) in rat cultured primary sensory neurons. A proportion of cells (466/1940, 24%) exhibited 18:0 LPC-evoked Ca^2+^ transients at 3 to 30 μM applied for 30 seconds (Figs. 2C and D). The number of neurons responding to 18:0 LPC exhibited a significant concentration-dependent increase (Fig. 2D; Fischer exact test, *P* < 0.0001). However, the average absolute amplitudes of the 18:0 LPC-evoked responses were not different from each other at 3 to 30 μM (Fig. 2E; ANOVA followed by Bonferroni post hoc test, *P* = 0.541, n = 90, 145, 231 at 3, 10 and 30 μM, respectively). Interestingly, 18:0 LPC induced a concentration-dependent increase in the amplitude of the KCl-evoked responses (Supplemental Fig. 6, available at http://links.lww.com/PAIN/B664; ANOVA followed by Bonferroni post hoc test, *P* ≤ 0.0001 between values at 3 and 30 μM, and 10 and 30 μM; n = 621, 620, 699 at 3, 10 and 30 μM, respectively).

Subsequently, we ascertained if 18:0 LPC-responding primary sensory neurons were nociceptive in function by assessing responsiveness of the cells to 500 nM capsaicin, a compound known to activate the great majority of nociceptive primary sensory neurons (Fig. 2F). A large proportion of 3 to 30 μM 18:0 LPC-responding cells also responded to 500 nM capsaicin (322 of 372 (86.6%); Figs. 2F and G). However, a proportion of capsaicin-responding neurons did not respond to 18:0 LPC (477 of 799, 59.7%, Figs. 2F and G). The proportion of cells that responded to both 18:0 LPC and capsaicin increased with escalating 18:0 LPC concentrations (3 μM: 16/256 (6.25%); 10 μM: 90/528 (17.05%); 30 μM: 216/747 (28.92%); Fischer exact test, *P* < 0.0001), whereas the proportion of 18:0 LPC-only-responding cells did not increase in response to escalating concentrations of 18.0 LPC (3 μM: 5/256 (1.95%); 10 μM: 19/528 (3.6%); 30 μM: 26/747 (3.48%); Fischer exact test, *P* > 0.05; Fig. 2G). Intriguingly, the overall proportion of cells responding to capsaicin also exhibited an apparent 18:0 LPC concentration-dependent increase (3 μM: 88/256 (34.38%); 10 μM: 252/528 (47.73%); 30 μM: 459/747 (61.46%); Fischer exact test, 3-10 μM *P* = 0.0004; 10-30 μM *P* < 0.0001; Fig. 2G). These findings collectively indicate that 18:0 LPC has multiple excitatory effects^43^ in primary sensory neurons; 18:0 LPC sensitises voltage-gated Ca^2+^ channels and the transient receptor potential ion channel (TRP), vanilloid subfamily, member 1 (V1; TRPV1), and activates molecule(s) that lead to an increase in the intracellular Ca^2+^ concentration. Previous findings^15,46,53,61,64,81^ as well as our query of the similarity ensemble approach (Supplemental Table 2, available at http://links.lww.com/PAIN/B664)^36^ show that LPCs—including 18:1 LPC—may indeed interact with several membrane molecules, including G protein–coupled receptors, some of which, such as LPA receptor 5, sensitise TRPV1.^34,38^

### 3.6. 18:0 lysophosphatidylcholine sensitises the capsaicin receptor TRPV1 but has an unspecific effect on the membrane of human embryonic kidney cells

**Figure 3.**
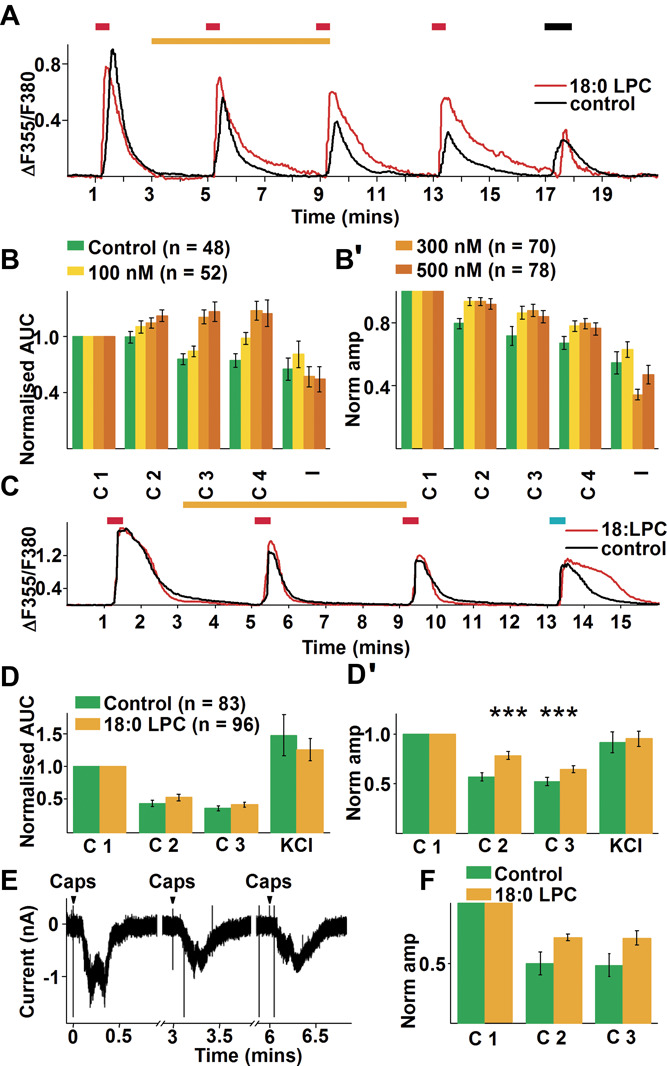
18:0 LPC sensitises TRPV1 in HEK293T cells and DRG neurons. (A) Recordings of changes in intracellular Ca^2+^ concentration to 30 nM capsaicin (black trace) and 30 nM capsaicin together with 18:0 LPC (red trace) in TRPV1-HEK293T cells. Red, yellow, and black bars above the recordings indicate 30 nM capsaicin, 18:0 LPC, and 60 mM KCl application, respectively. (B) Average AUC of control (green) and responses with various concentrations of 18:0 LPC (yellow, mustard, brown). (B′) Average amplitudes of capsaicin-evoked responses in control (green), and under various concentrations of 18:0 LPC (yellow, mustard, brown). (C) Recording of changes in intracellular Ca^2+^ concentration in cultured rat primary sensory neurons with 30 nM capsaicin (black) and capsaicin with 18:0 LPC (red). Red, yellow, and black bars above the recordings indicate 30 nM capsaicin, 500 nM 18:0 LPC, and 60 mM KCl application, respectively. (D) Average AUC of control (green) and responses with 500 nM 18:0 LPC (yellow). (D′) Average amplitudes of capsaicin-evoked responses in control (green), and capsaicin-evoked responses with 500 nM 18:0 LPC (yellow). (E) Whole-cell voltage clamp recordings of capsaicin-evoked currents in cultured primary sensory neurons. (F) Average amplitudes of capsaicin-evoked responses in control (green), and capsaicin-evoked responses with 500 nM 18:0 LPC (yellow). ***P < 0.0001. AUC, area under the curve; DRG, dorsal root ganglia; LPC, lysophosphatidylcholine.

To corroborate the sensitising effect of 18:0 LPC on TRPV1 further, we applied 18:0 LPC to nontransfected and human TRPV1-transfected human embryonic kidney 293 T (HEK) cells. Unexpectedly, 18:0 LPC induced an increase in [Ca^2+^]_i_ concentration of nontransfected HEK cells in a manner only partially dependent on the presence of Ca^2+^ in the extracellular buffer (Supplemental Fig. 7, available at http://links.lww.com/PAIN/B664). We also observed that a higher 18:0 LPC concentration shortened the delay between onset of 18:0 LPC application and initiation of the Ca^2+^ transient (Supplemental Fig. 7, available at http://links.lww.com/PAIN/B664). The 18:0 LPC-induced Ca^2+^ transients were associated with a significant change in cell shape; angular cells became rounded and, following an apparently irreversible Ca^2+^ load, cells ruptured (Supplemental Fig. 7, available at http://links.lww.com/PAIN/B664). However, limiting exposure of nontransfected and transfected HEK cells to 100 to 500 nM 18:0 LPC for ∼10 minutes prevented any such detrimental effects within the recording timeframe (Figs. 3A, B and B′).

At 100 to 500 nM, we found that 18:0 LPC induced a concentration-dependent increase in the AUC of the 30 nM capsaicin pulse-evoked responses and offset the desensitising effect of capsaicin assessed by the response amplitude (2-way repeated-measures ANOVA, effect of repeated capsaicin application *P* < 0.0001; effect of LPC concentration *P* < 0.0001; effect of repeated capsaicin applications × effect of LPC concentration *P* < 0.0001; Figs. 3A, B and B′). Notably, the sensitising effect outlasted the removal of 18:0 LPC application by at least 4 minutes (Figs. 3A, B and B′).

The effect of 500 nM 18:0 LPC on primary sensory neurons was less pronounced than on TRPV1-HEK cells, as neurons exhibited a modest but significant increase in the normalised amplitude of 30 nM capsaicin-evoked responses (2-way repeated-measures ANOVA, effect of LPC concentration *P* < 0.0001; Figs. 3C and D), although not their AUC. Results of whole-cell voltage-clamp recordings supported a sensitising effect of 18:0 LPC on capsaicin-evoked responses, although the apparent disruptive effect of 18:0 LPC on the gigaseal prevented collecting enough recordings for meaningful statistical comparison (Figs. 3E and F).

### 3.7. 18:0 LPC modifies heat-evoked responses in primary sensory neurons

**Figure 4.**
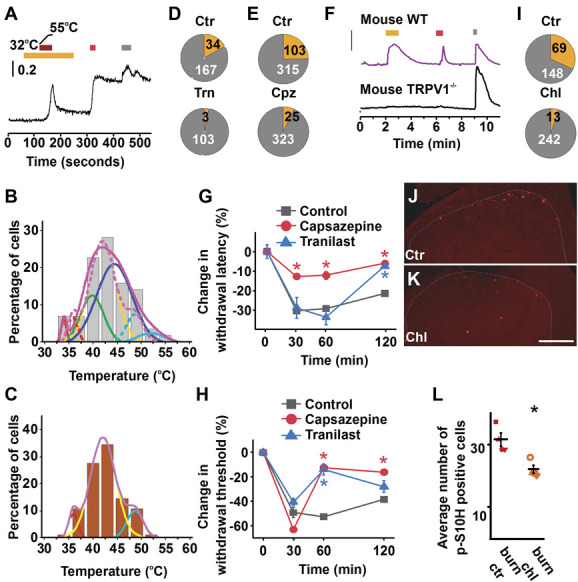
Increased lateral pressure by 18:0 LPC activates TRPV1 and TRPV2 and contributes to burn injury-induced pain. (A) Heat stimulation (dark red bar) increases [Ca^2+^]_i_ in a group of rat cultured primary sensory neurons. Yellow, red, and grey bars above the recording indicate 18:0 LPC (1 μM), capsaicin (500 nM), and KCl (60 mM) application, respectively. (B) Heat threshold distribution in the absence of 18:0 LPC. Solid lines indicate results of “multiple peak fit” function (Origin) on normally distributed components. Dotted lines indicate results of “multiple peak fit” function (Origin) on normally distributed components in the presence of 1 μM 18:0 copied from (C). (C) Heat threshold distribution in the presence of 1 μM 18:0 LPC. Solid lines indicate results of “multiple peak fit” function (Origin) on normally distributed components in the presence of 1 μM 18:0 LPC. (D–E) The TRPV2 and TRPV1 antagonists Trn (D) and capsazipeine (E), respectively, reduce the proportion of 18:0 LPC-responding neurons (yellow pie). Ctr, Trn (75 μM) Cpz (10 μM). (F) Deletion of TRPV1 reduces the 18:0 LPC (30 μM)-induced increase in the [Ca^2+^]_i_. Yellow, red, and grey bars above the recordings indicate 18:0 LPC, capsaicin (500 nM), and KCl (60 mM) applications, respectively. (G–H) Cpz (100 µL of 10 μM) and Trn (100 µL of 600 μM) (intraplantar) reduce hypersensitivity to heat (G) and mechanical (H) stimuli. (I) Chl reduces the proportion of 18:0 LPC-responding neurons (yellow pie). Ctr, Chl (75 μM). (J and K) Chl (50 mg/kg, i.p.) reduces the number of p-S10H3-expressing spinal cord neurons. Scale bar = 200 μm. (L) Average number of p-S10H3-expressing nuclei in the spinal dorsal horn. Ctr, Chl (50 mg/kg, i.p), *P < 0.05. Ctr, control; Chl, chlorpromazine; Cpz, capsazepine; LPC, lysophosphatidylcholine; Trn, tranilast.

TRPV1 is a stimulus integrator which, in addition to capsaicin is also responsive to a series of other activators including noxious heat with a threshold of >∼42°C.^12,55,77^ Via stimulus integration, the 18:0 LPC-induced TRPV1 sensitisation may reduce TRPV1's heat threshold, leading to a burning pain experience immediately after 18:0 LPC acts on primary sensory neurons.^55^ In the longer term, such sensitisation may significantly contribute to heat hyperalgesia, as TRPV1 is pivotal for the development of hypersensitivity to heat associated with tissue inflammation.^10,17,68^ We therefore assessed heat-evoked responses of cultured primary sensory neurons with and without (control) pretreatment with 1 μM 18:0 LPC, which does not induce excitation of sensory neurons per se (Fig. 4A). In control conditions, a heat ramp from 32 to 55°C induced responses in 57 of 184 neurons (30.98%) with heat thresholds defining 4 groups of cells (peak temperature threshold of the groups: 33.9, 39.8, 44.4, and 52°C with a cumulative threshold of 41.8°C; Fig. 4B).

In addition to TRPV1, primary sensory neurons express several other heat transducers with different temperature thresholds^11,68^ and various heat transducers often exhibit coexpression.^2,68,72,82,85^ While the response profile cannot therefore necessarily be attributed to a single heat transducer, the fitting curve with the highest peak threshold (52°C) is consistent with the previously reported heat threshold of the “high threshold noxious heat transducer” transient receptor potential ion channel, vanilloid subfamily, member 2 (TRPV2; Fig. 4B), and a significant proportion of TRPV2 expressing cells appear to have no other heat transducers.^2,11,12,56,59^ Furthermore, the fitting curve with the second highest peak (44.4°C) appears consistent with cells expressing TRPV1, which is often coexpressed with the transient receptor potential ion channel, melastatin subfamily member 3 and, to some extent, with TRPV2 (Fig. 4B).^52,85^

While pretreatment of cells with 1 μM 18:0 LPC did not increase the overall number of heat-responding neurons (142 out of 441 (32.2%), Fischer exact test, *P* = 0.78), it did change the distribution of heat thresholds; they formed 3 rather than 4 groups (Fig. 4C; 36, 42 and 48.6°C, with a cumulative threshold of 41.9°C). While the cumulative threshold appeared identical, the 2 higher thresholds were reduced by 2 and 4°C, respectively, by 1 μM 18:0 LPC (Fig. 4C). These results suggest that 18:0 LPC modifies heat responses of primary sensory neurons, which includes an apparent reduction in the heat activation threshold of TRPV2 and possibly of TRPV1.

### 3.8. 18:0 lysophosphatidylcholine activates TRPV1 and TRPV2

Next, we explored the ion channels involved in the 3 to 30 μM 18:0 LPC-induced increase in intracellular Ca^2+^ concentration. The capsaicin responsiveness of the majority of 18:0 LPC-responding cells and the 18:0 LPC-induced shift in heat threshold distribution suggested that 18:0 LPC may, in addition to sensitising, activate the 2 noxious heat-sensitive TRP channels, TRPV1 and TRPV2. Therefore, before applying 30 μM 18:0 LPC, we pretreated cultured primary sensory neurons with either capsazepine (10 μM) or tranilast (75 μM), TRPV1 and TRPV2 antagonists, respectively.^8,29,71^ Pretreatment of neurons with tranilast significantly reduced the proportion of neurons responding to 18:0 LPC (control: 34/201 (16.92%); tranilast: 3/106 (2.83%); Fischer exact test, *P* < 0.0001; Fig. 4D). Pretreatment with capsazepine also significantly reduced the number of 18:0 LPC-responding neurons (control: 103/418 (24.64%); capsazepine: 25/348 (7.18%); Fischer exact test, *P* < 0.0001; Fig. 4E). To corroborate this latter finding, we also studied the effect of 30 μM 18:0 LPC on primary sensory neurons isolated from WT and TRPV1^−/−^ mice (Fig. 4F). As expected, 18:0 LPC activated significantly fewer neurons in cultures prepared from DRGs of TRPV1^−/−^ than from DRGs of WT mice (WT: 124/280 (44.29%); TRPV1^−/−^: 1/234 (0.44%) Fischer exact test, *P*=<0.0001; Fig. 4F).

### 3.9. Blocking TRPV1 or TRPV2 reduces 18:0 lysophosphatidylcholine-evoked pain-related behaviour

As 18:0 LPC induces sensitising and excitatory effects on TRPV1 and TRPV2 in primary sensory neurons, we assessed the effect of blocking these ion channels on 18:0 LPC-induced hypersensitivity to heat and mechanical stimuli (Figs. 4G and H). We injected 100 µL of capsazepine or tranilast, respectively, at 10 or 600 µM into the plantar skin of the right hind paw 15 minutes before injection of 0.4% LPC. Consistent with in vitro findings, both capsazepine and tranilast significantly reduced the 0.4% 18:0 LPC-evoked pain-related behaviour (repeated measurement two-way mixed design ANOVA, Tukey post hoc test, n = 6, *P* < 0.05 for both drugs; Figs. 4G and H). While capsazepine reduced 18:0 LPC-induced heat hyperalgesia within 30 minutes, tranilast had a significant effect only after 2 hours (Fig. 4G). However, both capsazepine and tranilast reduced 18:0 LPC-induced mechanical allodynia by 60 minutes (Fig. 4H).

### 3.10. Blocking increased lateral membrane pressure reduces the number of 18:0 lysophosphatidylcholine-responding primary sensory neurons

While LPCs directly activate a number of receptors,^15,23,46,58^ they may also induce their effects by increasing lateral pressure in the membrane.^47^ Our findings that 18:0 LPC changes the shape of HEK cells (Supplemental Fig. 7, available at http://links.lww.com/PAIN/B664) and disrupts the gigaseal during electrophysiology support the notion that 18:0 LPC incorporates into the outer leaflet of the membrane and increases lateral pressure on membrane proteins. Consistent with this, pretreatment of primary sensory neurons with chlorpromazine (75 μM), a conical amphipathic molecule known to inhibit increases in lateral pressure,^14^ significantly reduced the 30 μM 18:0 LPC-evoked increase in [Ca^2+^]_i_ (control: 69 of 217 (31.8%), chlorpromazine: 13 of 255 (5.1%), Fischer exact test, *P* < 0.0001; Fig. 4I). These findings indicate that changes in lateral pressure by 18:0 LPC significantly contribute to its excitatory effect in nociceptive primary sensory neurons.

### 3.11. Chlorpromazine reduces 18:0 lysophosphatidylcholine-induced spinal nociceptive processing

To determine whether the excitatory effect of 18:0 LPC and other molecules in burn skin, which are able to increase lateral pressure, contribute to pain development in burn injury, we explored chlorpromazine's effect on burn injury-induced nociceptive processing through assessing the spinal expression of phosphorylated serine 10 of histone 3 (p-S10H3), an indicator of activation of superficial spinal dorsal horn neurons in tissue injuries including burn injury and subsequent inflammatory pain conditions.^79,83^ Consistent with previous findings, burn injury of the hind paw resulted in phosphorylation of S10H3 in a group of superficial spinal cord neurons (Supplemental Fig. 8, available at http://links.lww.com/PAIN/B664; Fig. 4J). Chlorpromazine (50 mg/kg i.p.) administered 10 minutes after injury significantly reduced the number of cells expressing p-S10H3 in the superficial dorsal horn at 1 hour post injury (n = 4; *P*= 0.0085, Student *t* test; Figs. 4J–L).

## 4. Discussion

Our findings demonstrate, for the first time, that 18:0 LPC is significantly and consistently altered in skin following burn injury; 18:0 LPC's level is significantly increased within an hour in an animal model of burn injury and remains elevated for days after the injury in human burn skin. The elevated concentration of 18:0 LPC in burn skin is consistent with the formation of LPCs during oxidative stress, a characteristic feature of burn injury.^1,42,57^ 18:0 LPC, similarly to other LPCs, is a proinflammatory molecule^41,44^; hence, its increase in burn skin is further consistent with local and systemic inflammatory reaction,^39,42^ both among the most important hallmarks of burn injury.

Increased LPC, including 18:0 LPC, levels have been found in various tissue injuries such as ischemia (myocardial, renal and brain) and trauma (corneal and surgical injuries) and contribute to human diseases including the development of inflammation, atherosclerotic plaques, insulin resistance, and pain-related behaviour such as nocifensive behaviour and mechanical hypersensitivity.^35,39,41,44,46,49,61,64,65,76,80^ Our findings demonstrate that, in addition to immediate pain and development of mechanical allodynia, 18:0 LPC also induces development of heat hyperalgesia. We also demonstrate that TRPV1 and TRPV2, 2 heat-sensitive ion channels, are involved both in immediate activation of primary sensory neurons and development of hypersensitivities to mechanical and heat stimulation by 18:0 LPC.

We have demonstrated the effect of 18:0 LPC on TRPV2 using tranilast, an agent that was originally introduced to treat allergic reactions.^16^ While tranilast's molecular targets have not been fully elucidated, it has been used to inhibit TRPV2-mediated responses.^16,29^ Although, TRPV2 is expressed by a lower proportion of primary sensory neurons than TRPV1,^2^ tranilast produced a higher reduction in the proportion of 18:0 LPC-responding neurons, than the TRPV1 antagonist capsazepine.^8^ We have not addressed this discrepancy in our study and can therefore only speculate that tranilast's inhibitory effect on protein kinase C activity, which is needed for TRPV1 responsiveness, could have produced the higher than expected reduction in 18:0 LPC-responding neurons.^55,66^ Nevertheless, the involvement of TRPV1 and TRPV2 in 18:0 LPC-evoked primary sensory neuron activation and development of hypersensitivities is in agreement with previous findings that various species of LPCs act through these channels.^12,53,61^

In addition to TRPV1 and TRPV2, LPCs, including 14:0 and 16:0 LPC, act on other ion channels including two-pore domain mechanogated potassium channels, the TRP melastatin subfamily, member 8 (TRPM8), ankyrin subfamily, member 1 (TRPA1), and vanilloid subfamily, member 4.^5,15,45,51,54,61,81^ While LPCs bind to some of these ion channels, the LPC-evoked activation of the two-pore domain mechanogated potassium channels and TRPM8 is blocked by chlorpromazine, which, in addition to interacting with various receptors, also acts as a negative membrane curvature agent.^15,30,31,48,70,81^ Presently, we demonstrate that chlorpromazine, similar to capsazepine and tranilast, significantly reduces 18:0 LPC-evoked activation of primary sensory neurons. This is consistent with increased lateral pressure by 18:0 LPC activating TRPV1 and TRPV2 in primary sensory neurons.

In addition to blocking 18:0 LPC-induced activity of primary sensory neurons, here we also demonstrate that chlorpromazine reduces the number of spinal dorsal horn neurons exhibiting p-S10H3 expression, which up-regulates very quickly following tissue injury/inflammation.^75,79,84^ Spinal p-S10H3 expression shows a clear association with inflammatory pain and sensitivity to noxious heat; blocking or deleting mitogen- and stress-activated kinases 1 and 2, the writers for this epigenetic tag,^78^ respectively, reduces nocifensive behaviour induced by subcutaneous formalin injection into the paw or blocks the development of inflammatory heat hyperalgesia induced by subcutaneous injection of the inflammation-inducing agent, carrageenan into the paw.^75,79^ Furthermore, blocking phosphorylation of S10 in H3 in spinal cord dynorphinergic neurons, which constitute the majority of neurons exhibiting up-regulation in p-S10H3 after burn injury,^84^ significantly reduces responsiveness of mice to noxious heat.^52^ Importantly, although chlorpromazine is an antagonist/inverse agonist of a series of dopamine, serotonin, and α2 adrenergic receptors, which are expressed in various components of spinal dorsal horn circuitries and supraspinal neurons that send fibres into, and modulate nociceptive processing in, the spinal dorsal horn, it has no analgesic effect in tissue injury.^6,9,26,28,88^ Therefore, the only plausible explanation for the effect of chlorpromazine on p-S10H3 expression is that, following chlorpromazine injection, the spinal nociceptive input from primary sensory neurons was reduced. That reduction is highly likely because of the inhibitory effect of chlorpromazine on the 18:0 LPC-induced increase in lateral pressure of the cytoplasmic membrane and subsequent activation of TRPV1, TRPV2, and possibly other TRP channels.^14^

In summary, using novel approaches, we have discovered that LPCs, specifically 18:0 LPC, significantly contribute to the development of mechanical and heat hyperalgesia in burn injury. This pain-inducing effect is not specific to burn injury but rather is likely to be an important mechanism for developing hypersensitivities in several tissue injuries and subsequent inflammatory states. Importantly, our metabolomic and lipidomic data, together with previous findings on the algogenic effects of many inflammatory mediators, indicate that LPCs act alongside a myriad of other agents to induce the development of hypersensitivities to thermal and mechanical stimuli associated with tissue injuries and inflammation. Hence, elucidating common features of downstream signalling by influential algogenic molecules such as LPCs offers an exciting avenue for identifying ways to reduce pain in tissue injury and inflammation.

## Conflict of interest statement

The authors have no conflicts of interest to declare.

## Appendix A. Supplemental digital content

Supplemental digital content associated with this article can be found online at http://links.lww.com/PAIN/B664.

## Supplementary Material

SUPPLEMENTARY MATERIAL
