# Supplementary material for: Elevated 18:0 lysophosphatidylcholine contributes to the development of pain in tissue injury
Source: Pain. 2022 Jun 7;164(2):e103–15. doi: 10.1097/j.pain.0000000000002709 (PMC9833116; doi:10.1097/j.pain.0000000000002709)
Supplement: SUPPLEMENTARY MATERIAL [file jop-164-e103-s001.pdf]

SUPPLEMENTARY MATERIALS

Elevated 18:0 lysophosphatidylcholine contributes to the development of pain in burn injury

Dominic Anthony Friston[1], Joshua Cuddihy[1,2], Jessica Luiz[1], An Hoai Truong[1], Laptin Ho[1], Meirvaan Basra[1], Peter Santha[3], Orsolya Oszlacs[3], Joao de Sousa Valente[4], Tim Marczylo[5], Sini Junttila[6], Helen Laycock[1]; Declan Collins[2], Marcela Vizcaychipi[1,2], Attila Gyenesei[7], Zoltan Takats[8], Gabor Jancso[3], Elizabeth Want[8]†, Istvan Nagy[1]†[*]

1. Nociception Group, Division of Anaesthetics, Pain Medicine and Intensive Care, Department of Surgery and Cancer, Imperial College London, Chelsea and Westminster Hospital, 369 Fulham Road, London, SW10 9NH, UK.
2. Department of Anaesthetics, Chelsea and Westminster NHS Trust, 369 Fulham Road, London, SW10 9NH, UK.
3. Department of Physiology, University of Szeged, 10 Dom ter, Szeged, H-6720, Hungary.
4. Section of Vascular Biology and Inflammation, School of Cardiovascular Medicine and Research, BHF Cardiovascular Centre of Research Excellence, King's College London, Franklin-Wilkins Building, London, SE1 9NH, UK.
5. UK Health Security Agency, Radiation, Chemical and Environmental Hazards, Fermi Avenue, Harwell Business Park, Chilton. OX11 0RQ.
6. Turku Bioscience Centre, University of Turku, Tykistökatu 6, Turku, 20520, Finland.
7. Szentagothai Research Centre, University of Pecs, Ifjusag str. 20, Pécs, H-7624, Hungary.
8. Biomolecular Medicine, Department of Metabolism, Digestion and Reproduction, Imperial College London, Exhibition Road, SW7 2AZ, London, UK.

*Corresponding author:
Istvan Nagy, MD, PhD, DSc
Nociception Group
Division of Anaesthetics, Pain Medicine and Intensive Care
Department of Surgery and Cancer
Faculty of Medicine
Imperial College London
Chelsea and Westminster Hospital
369 Fulham Road
London
SW10 9NH
United Kingdom
+44-020- 3315-8897
i.nagy@imperial.ac.uk

†equal contribution

One sentence summary: 18:0 lysophosphatidylcholine is a major pain-inducing molecule in injured tissues.

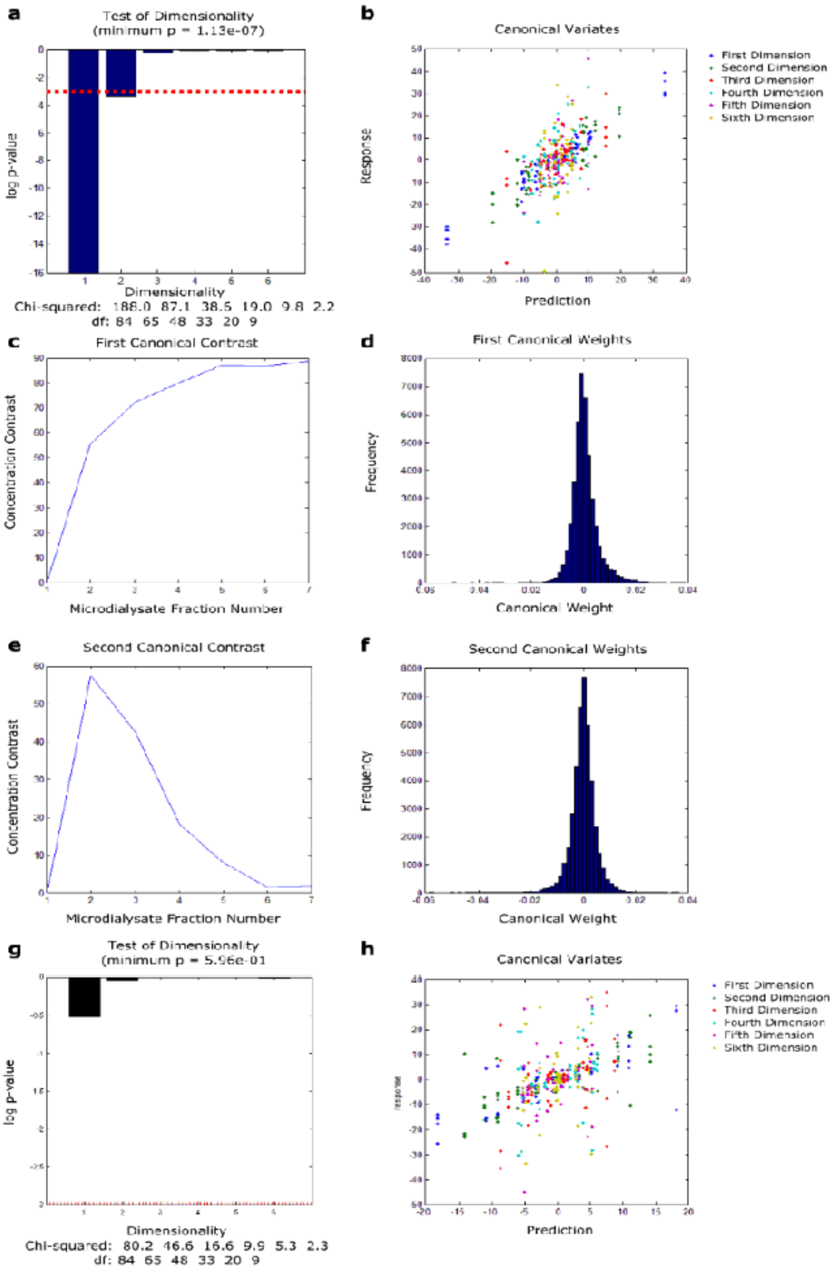

Supplementary Figure 1
Canonical variates analysis of ESI+ UPLC-MS data. (a) expresses log p-values, and respective chi squared distributions and degrees of freedom on which they are based, where the red dash line indicates statistical significance. Each p-value represents a potential dimension of the metabolite concentrations' dependency on the explanatory variables; practically, each represents a different temporal profile conserved over the time courses of a subset of metabolites. (b) degree of correlation between mixtures of variables subtending the correlation whose significance is reported in the first subplot; a high correlation indicates a consistent temporal profile across explanatory variables. Where (a) indicates the significance of two temporal profiles, constituting a two-dimensional burn effect, (c) expresses the contrast in concentration trajectories in the first dimension; it consists of a sustained increase in difference between burn and control concentrations over time. (d) histogram of metabolite contributions to the canonical correlation, and thereby their similarities to

the temporal profile in (c); the skew suggests that a small number of metabolites contribute heavily to the treatment effect (site by time interaction), while most contribute negligibly. (e) and (f) respectively express the same as (c) and (d) for the second dimension of the burn effect; it is characterised by a transient increase in difference between burn and control concentrations. (g) and (h) respectively present p-values and variable mixture correlations for the same analysis of the data following randomisation of its order within individual metabolite time series; the lack of a significant result here, with treatment effects removed by randomisation, indicates that the significant results for the un-randomised data does not result from violations of distributional assumptions.

CVA: The effects of interest correspond to interaction between the microdialysis site (with two levels: burn and control) and the time post-injury (with seven levels: one pre- and six post-burn microdialysate fractions), observed as a difference in signal intensities between the burn and control microdialysates over time post-burn. This was specified in a design matrix encoding the effects of time at each site as experimental factors and confounds represented by a constant term for each set of longitudinal data. Fixed effects analysis pooling over the four subjects was used. Singular value decomposition was applied to decrease the dimensionality of the metabolomics data to 25% of the number of observations.

Significance was determined via transformations of Wilke's Lambda in the form of multiple p-values reflecting the potential multidimensional dependence of the feature signal intensities on the interaction between microdialysis site and time. Each p-value represents the significance of a contrast between burn and control concentrations over time expressed by a subset of metabolites; the contribution of each metabolite to that profile is quantified by its canonical weight, as is expressed in a canonical vector (the collective of all canonical weights for a given dimension). For significant dimensions, metabolites were hence ranked by canonical weight as a means of identifying burn-altered discriminants.

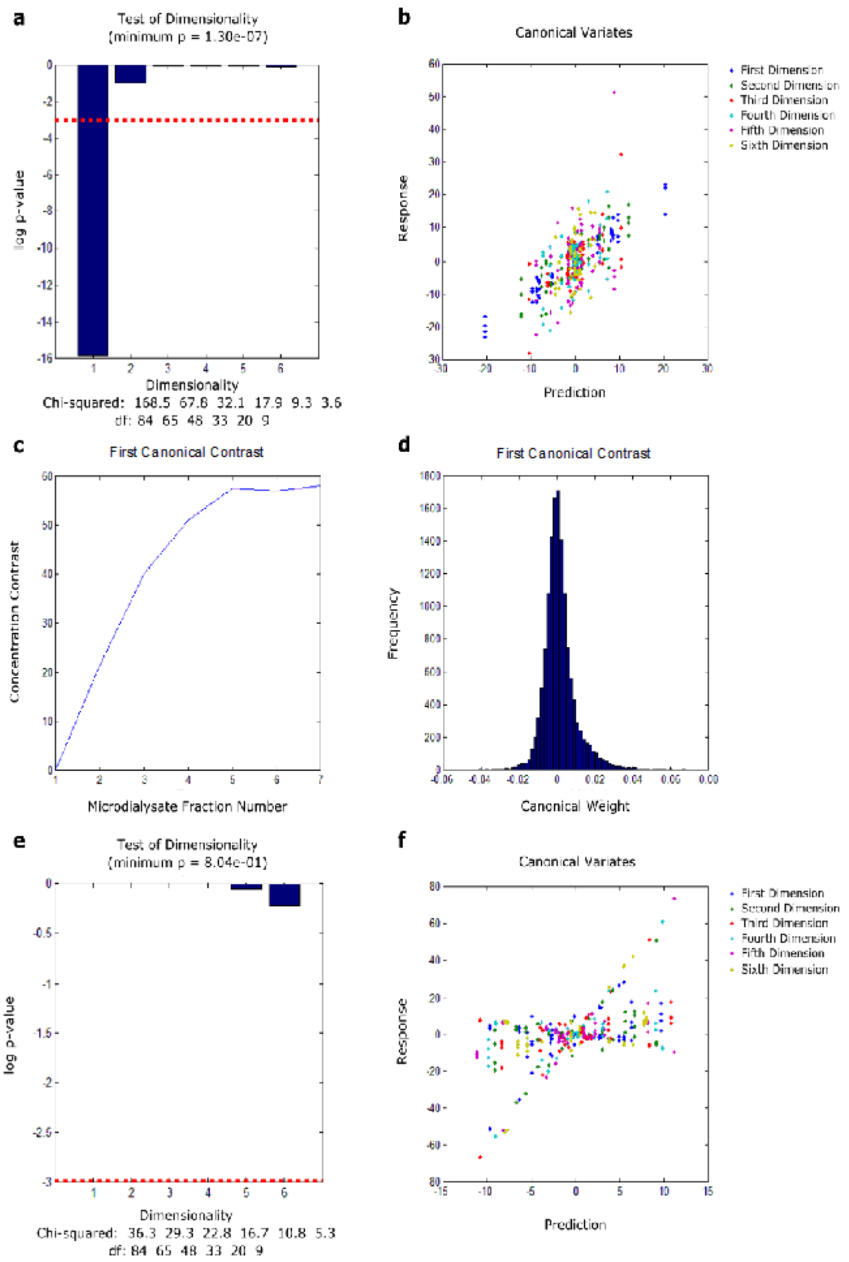

Supplementary Figure 2

Canonical variates analysis of ESI- UPLC-MS data. (a) expresses log p-values, and respective chi squared distributions and degrees of freedom on which they are based, where the red dash line indicates statistical significance. Each p-value represents a potential dimension of the metabolite concentrations' dependency on the explanatory variables; practically, each represents a different temporal profile that is conserved over the time courses of a subset of metabolites. (b) indicates the degree of correlation between mixtures of variables subtending the correlation whose significance is reported in the first subplot; a high correlation indicates a consistent temporal profile across explanatory variables. Where (a) indicates the significance of one temporal profile, constituting a one-dimensional burn effect, (c) expresses the contrast in concentration trajectories in this dimension; it consists of a sustained increase in difference between burn and control concentrations over time. (d)

histogram of the metabolite contributions to the canonical correlation, and thereby their similarities to the temporal profile in (c); the skew suggests that a small number of metabolites contribute heavily to the treatment effect (site by time interaction), while most contribute negligibly. (e) and (f) respectively present p-values and variable mixture correlations for the same analysis of the data following randomisation of its order within individual metabolite time series; the lack of a significant result here, with treatment effects removed by randomisation, indicates that the significant results for the unrandomised data does not result from violations of distributional assumptions.

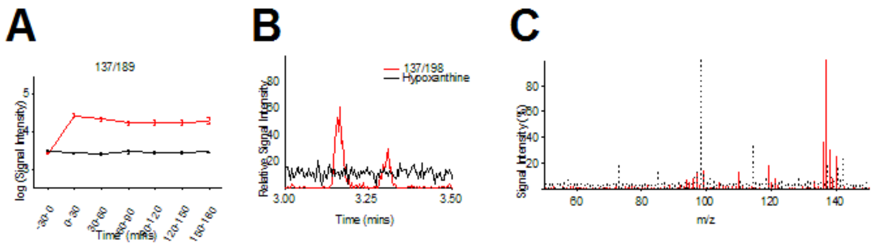

Supplementary Figure 3
***m/z* RT 137/198 does not share a structure with hypoxanthine.**
(A) Temporal change of *m/z* RT 137/198 in control (black) and burn (red) samples.
(B) Comparative elution profiles and (C) fragmentation spectra of feature *m/z* RT 137/198 (red) and a standard preparation of hypoxanthine (black) indicate that the compounds did not similarly interact with the chromatography column or fragment upon collision in UPLC-MS/MS; the possibility that *m/z* RT 137/198's is hypoxanthine can therefore be excluded.

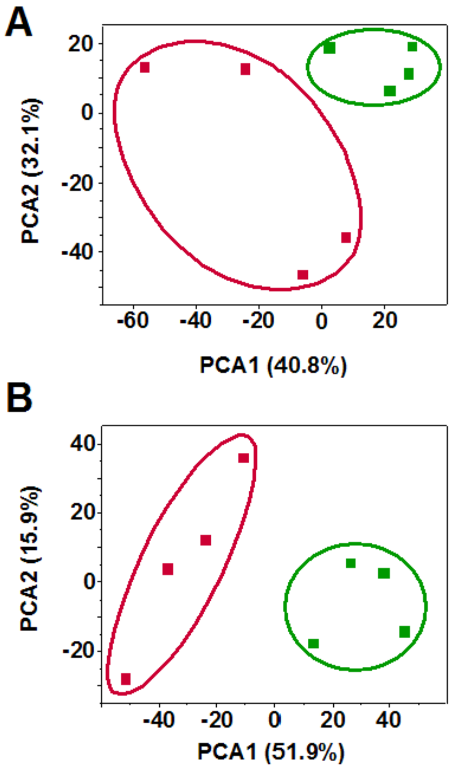

Supplementary Figure 4
Burn injury results in significant changes in the abundance of lipids in the human skin. (**A**) PCA score plot of burn (red) and control (green) skin samples based on a UPLC-MS lipidomics assay shows clear separation of samples in ESI- mode. (**B**) PCA scores plot of burn (red) and control (green) skin samples based on the same lipidomics assay also shows clear separation of samples in ESI+ mode. Percentage values indicate the contribution of first and second component to variance in both (**A**) and (**B**).

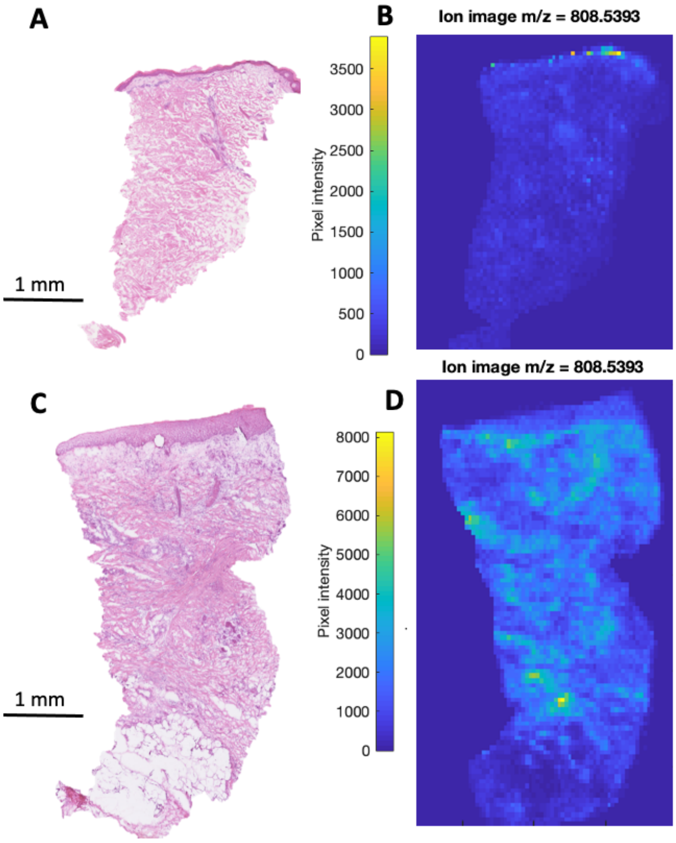

Supplementary Figure 5

The immediate precursor of 18:0 LPC, 18:0/18:2 phosphatidylcholine, is increased following burn injury in the human skin

(**A**) Microphotograph of haematoxylin and eosin stained section cut from control human skin biopsy. (**B**) DESI-MS image of *m/z* 808.582, which is a match for 18:0/18:2 phosphatidylcholine+Na+ (18:0/18:2 PC+Na+) in the section shown in (**A**). (**C**) Microphotograph of haematoxylin and eosin stained section cut from burn human skin biopsy. (**D**) DESI-MS image of *m/z* 808.582, which is a match for 18:0/18:2 phosphatidylcholine+Na+ (18:0/18:2 PC+Na+) in the section shown in (**C**).

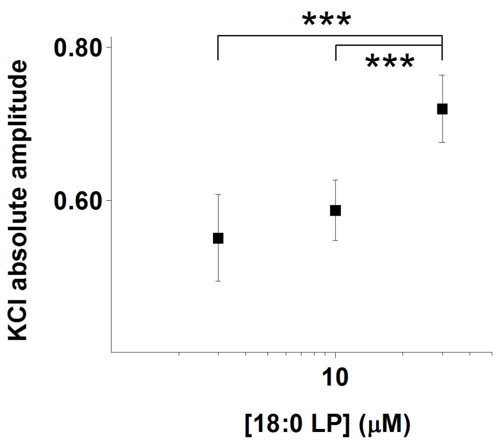

Supplementary Figure 6
18:0 LPC at 3-30μM induces a concentration-dependent increase in the amplitude of the KCl-evoked-responses.
Absolute amplitudes of Ca^{2+} transients measured during ratiometric calcium imaging of rat cultured primary sensory neurons. Asterisks indicate significant increase at 30μM when compared to 3μM or 10μM.

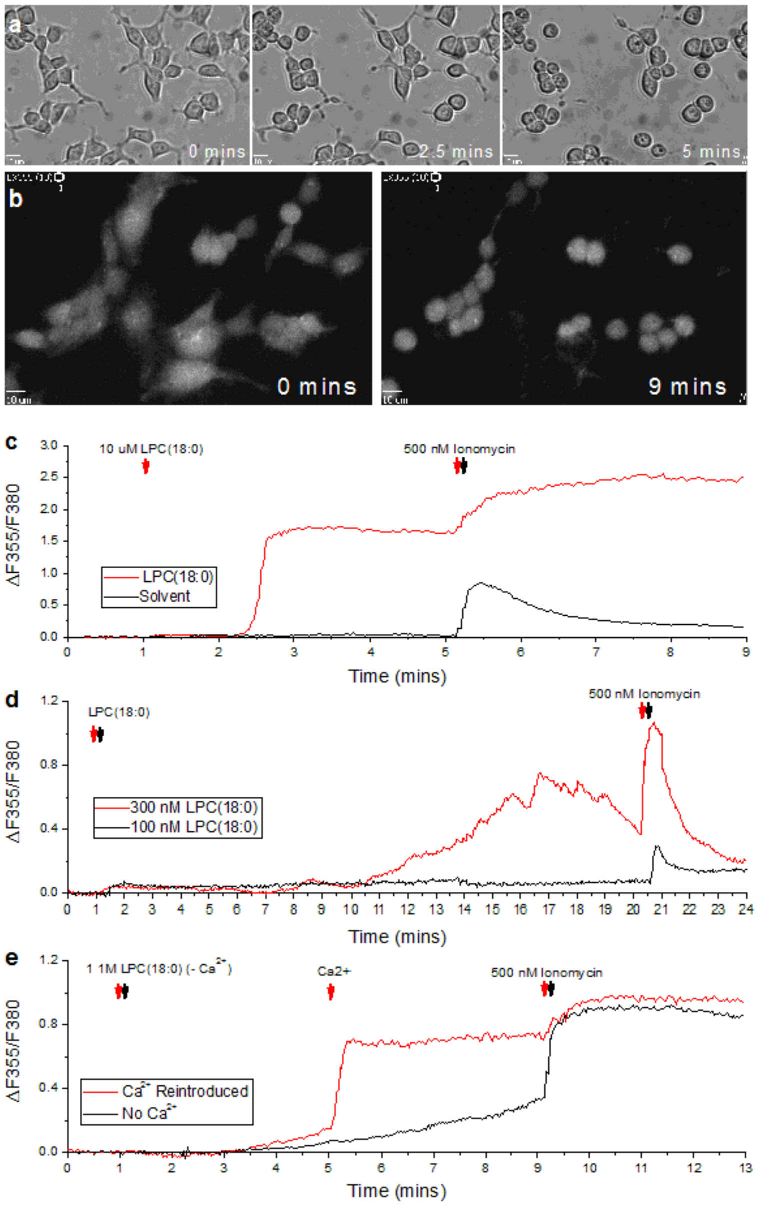

Supplementary Figure 7

18:0 LPC induces calcium influx, morphological changes and lysis in untransfected HEK293T cells.

(**a**) At a sufficient concentration and duration of exposure, 18:0 LPC induces cell rounding in and (**b**) lyses the membranes of HEK293T cells. (**c**) Prior to lysis, intracellular Ca^{2+} overload occurs within 2 min of 10μM 18:0 LPC exposure. (**d**) The non-specific deleterious membrane effects can be delayed or prevented at sufficiently low 18:0 LPC concentrations. (**e**) Intracellular Ca^{2+} overload is mediated by influx of extracellular calcium, as indicated in its preclusion by Ca^{2+} deprivation and its onset with reintroduction of extracellular Ca^{2+}.

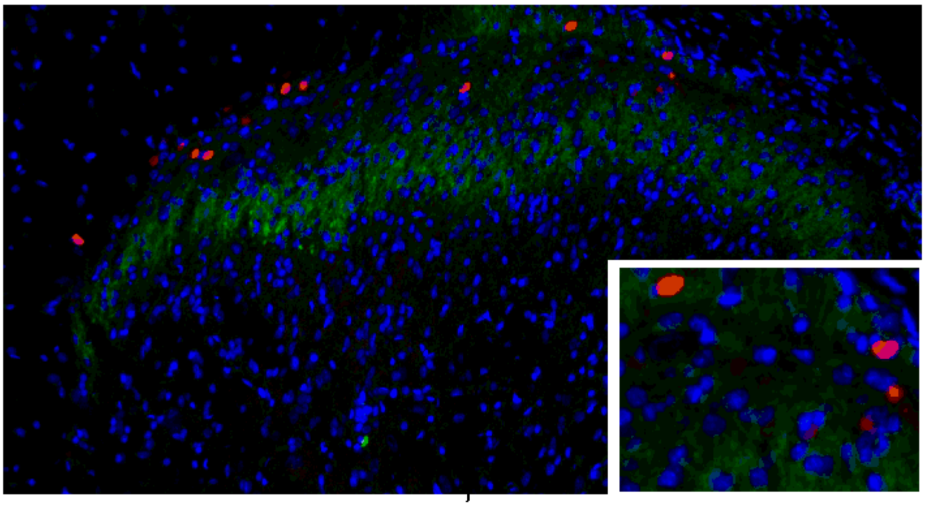

Supplementary Figure 8
Following burn injury, p-S10H3 (purple)-expressing nuclei are found predominantly in lamina I and II_o of the spinal dorsal horn.
Burn injury induces phosphorylation of S10 in histone H3 in the superficial spinal dorsal horn. We have shown previously that the p-S10H3-expressing nuclei are neuronal nuclei. Green shown isolectin B4 staining which indicates the ventral border for Lamina II_i. Blue colour is due to the nuclear stain diamidino-2-phenylindole (DAPI; blue).

Supplementary Table 1
Metabolomics UPLC and MS parameters.

Metabolomics UPLC parameters
Mobile Phase A =
Flow rate = 0.4ml/min

Time	Mobile Phase A	Mobile Phase B
0	99	1
1	99	1
3	85	15
6	50	50
9	5	95
10	5	95
12	99	1

Metabolomics MS parameters

Parameter	Positive mode	Negative Mode
Cone voltage	30V	30V
Capillary voltage	+1 KV	-1 KV
Source Temperature	120°C	120°C
Desolvation Temperature	450°C	450°C
Desolvation gas flow	900 L/h	900 L/h
Cone gas flow	50 L/h	50 L/h

Supplementary Table 2
Putative targets for 18:0 LPC predicted by the Similarity Ensemble Approach.

Target name	Species	P-Value	MaxTC
1-phosphatidylinositol 4,5-bisphosphate phosphodiesterase gamma-2	human	7.72E-10	0.37
Bifunctional epoxide hydrolase 2	mouse	9.82E-09	0.31
Cocaine esterase	human	1.08E-06	0.31
Dynamin-1	human	2.89E-12	0.29
Ectonucleotide pyrophosphatase/phosphodiesterase family member 2	human	9.81E-14	0.55
Fatty acid-binding protein, heart	human	1.61E-06	0.58
Lysophosphatidic acid receptor 1	mouse	1.71E-46	0.31
Lysophosphatidic acid receptor 1	human	1.16E-21	0.62
Lysophosphatidic acid receptor 2	human	7.47E-26	0.62
Lysophosphatidic acid receptor 3	human	1.93E-49	0.62
Lysophosphatidic acid receptor 4	human	4.19E-110	0.62
Lysophosphatidic acid receptor 4	mouse	2.14E-68	0.55
Lysophosphatidic acid receptor 5	human	2.59E-08	0.62
Lysophosphatidic acid receptor 6	human	3.60E-92	0.62
M-phase inducer phosphatase 2	mouse	2.64E-11	0.3
Mitochondrial carnitine/acylcarnitine carrier protein	human	5.39E-48	0.38
P-selectin	human	2.92E-06	0.4
Peptidyl-glycine alpha-amidating monooxygenase	human	2.40E-22	0.37
Phospholipase A2, membrane associated	mouse	1.20E-06	0.28
Probable G-protein coupled receptor 174	human	6.83E-168	0.54
Probable G-protein coupled receptor 34	human	2.64E-104	0.54
Probable G-protein coupled receptor 34	mouse	3.37E-20	0.48
Protein kinase C alpha type	rat	1.03E-07	0.29
Protein kinase C delta type	mouse	1.22E-08	0.38
Protein kinase C eta type	mouse	5.74E-21	0.4
Protein phosphatase 1A	human	1.20E-26	0.33
Putative inactive group IIC secretory phospholipase A2	human	2.69E-20	0.32
Putative P2Y purinoceptor 10	human	1.22E-188	0.54
Putative P2Y purinoceptor 10	mouse	2.22E-16	0.48
Sphingomyelin phosphodiesterase 2	rat	6.55E-08	0.31
Sphingosine 1-phosphate receptor 2	human	5.04E-07	0.34
Sphingosine 1-phosphate receptor 4	human	4.86E-06	0.3

Sphingosine 1-phosphate receptor 5	human	1.17E-08	0.33
Toll-like receptor 2	human	6.81E-67	0.39
Vascular endothelial growth factor A	human	3.71E-11	0.33
